# Clinical importance of preoperative red-cell volume distribution width as a prognostic marker in patients undergoing radical surgery for pancreatic cancer

**DOI:** 10.1007/s00595-021-02374-7

**Published:** 2021-09-15

**Authors:** Chao Dang, Min Wang, Tingting Qin, Renyi Qin

**Affiliations:** Department of Pancreatic-Biliary Surgery, Affiliated Tongji Hospital, Tongji Medical College, Huazhong University of Science and Technology, 1095 Jiefang Ave, Wuhan, 430030 China

**Keywords:** Red-cell volume distribution width, Prognostic marker, Periampullary carcinoma, Pancreaticoduodenectomy, Nutritional status

## Abstract

**Background and purpose:**

A new noninvasive biomarker is being sought to predict the prognosis of patients with pancreatic cancer. Red-cell volume distribution width (RDW), a descriptive parameter for erythrocyte variation, has been shown to have prognostic value for some tumor types. Our purpose was to assess the RDW value to predict the prognosis of patients with pancreatic cancer.

**Methods:**

The subjects of this retrospective study were 792 patients who underwent radical surgery for pancreatic cancer, divided into high-RDW and low-RDW groups based on receiver operating characteristic (ROC) curve analysis (15.6%). The controlling nutritional status (CONUT) score was used to assess preoperative nutritional status. Statistical analysis was conducted to investigate the differences between the high and low RDW groups, and to explore the possibility of the RDW being used as prognostic predictor for patients with pancreatic cancer.

**Results:**

The immune-nutritional status was worse in the high-RDW group than in the low-RDW group. The high-RDW group patients also had a poorer prognosis. Risk factor analysis showed that the RDW could be an independent risk factor for pancreatic cancer.

**Conclusions:**

The RDW is associated with immune-nutritional status in pancreatic cancer patients and can be used as an independent prognostic factor for their postoperative survival.

**Supplementary Information:**

The online version contains supplementary material available at 10.1007/s00595-021-02374-7.

## Introduction

Pancreatic cancer is one of the most aggressive malignant tumors and the fourth-leading cause of cancer-related death worldwide [1]. Currently, surgical resection is the only curative option for patients with this disease [2], but pancreatic surgery is complex and highly technical. Although pathological analysis is helpful for predicting prognosis, it is more important to establish preoperatively which patients could achieve long-term survival through surgery. Over the past decade, studies on tumor and inflammatory markers, circulating tumor cells, and gene signatures have shown the potential for the preoperative prediction of postoperative survival after pancreatic cancer surgery [3–8].

There has been increasing interest in identifying new noninvasive predictive biomarkers from among various hematological and serological parameters. Red-cell volume distribution width (RDW) is based on the width of the red blood-cell volume distribution curve, which reflects changes in the size of circulating red blood cells. The change in RDW is related to changes in the erythrocyte survival pattern, which indicates the derailment of erythropoiesis [9, 10]. The RDW, which is the main descriptive parameter of erythrocyte variation, is associated with poor prognosis in some diseases [11–13]. Previous studies have shown that the RDW may have diagnostic and prognostic value for a variety of tumor types, including lung cancer, liver cancer, prostate cancer, esophageal cancer, and chronic lymphocytic leukemia [14–17]. However, the effects of the preoperative RDW on the prognosis of patients with pancreatic cancer have rarely been reported. The purpose of this study was to investigate the association between the preoperative RDW and the prognosis of patients with pancreatic cancer after radical surgery. The relationship between RDW and nutrition is also discussed.

## Materials and methods

### Study population and patient selection

A single-center cohort of 792 pancreatic cancer patients who underwent radical surgery between January, 2011 and January, 2019 at the Institute of Biliary-Pancreatic Surgery, Tongji Hospital, Tongji Medical College, Huazhong Scientific and Technological University, was analyzed retrospectively. Patients with inadequate baseline data or missing primary outcome data were excluded from the analysis. Data were collected on patient characteristics, surgical details, morbidity and mortality, postoperative length of stay, and pathological outcomes. Preoperative examination included an appropriate imaging diagnosis to exclude distant metastases. Preoperative characteristics included age, sex, complications, body mass index (BMI), and American society of anesthesiologists (ASA) score. The CONUT score, which was considered to represent the immune-nutritional status, was calculated from the serum albumin concentration, total peripheral lymphocyte count, and total cholesterol level. Based on the original report by Ignacio et al. [18], the CONUT scoring system ranges from normal (0–1) to severe (9–12) (Online resource 1). Surgical details included operative time (from incision to wound closure), estimated blood loss, and transfusion volume (obtained from anesthesia records). Postoperative complications were classified according to international standards. The patients were followed up regularly in the first month after surgery, then every 3 months for 2 years, and every 6 months thereafter. The last follow-up time recorded was in December, 2019. Informed consent from patients was not required due to the retrospective design of the study. This study was approved by the review committee of the faculty of Tongji Medical College of the Huazhong University of Science and Technology.

### Definitions

Operative time was defined as the time from the skin incision or trocar insertion to complete skin closure. Intraoperative estimated blood loss was recorded by the anesthesiologist through a vacuum system. Postoperative hospital stay was defined as the days from surgery to discharge. Morbidity and mortality were defined as any complications or deaths during hospitalization or within 30 days of discharge after surgery. Readmission within 30 days after surgery was considered unplanned. Postoperative complications were evaluated according to the Clavien-Dindo (CD) classification system [19] and included postoperative pancreatic fistula (POPF) [20], delayed gastric emptying (DGE) [21], bile leakage [22], and postoperative bleeding [23].

### Statistical analysis

The main endpoint was overall survival (OS), which was calculated from the date of surgery to the date of death or last follow-up. Continuous variables were reported as means with standard deviations, or medians with interquartile range (IQR) and were compared using the student’s *t*-tests or Mann–Whitney tests. Categorical data are presented as frequencies (%) and compared using the Chi-square or Fisher’s exact tests. Survival analysis was performed using the Kaplan–Meier method to evaluate the survival time distribution. The log-rank test was used when indicated.

The cutoff values of RDW for predicting survival were calculated using receiver operating characteristic (ROC) curve analysis; then the RDW was dichotomized into low and high groups. Multivariate Cox regression analysis with stepwise adjusting covariates was performed to assess the relationship between high and low RDW concentrations and OS. The hazards ratio (HR) as well as 95% CI were calculated. To estimate the raw relationship of RDW and OS, the initial statistical model (Model 1) was used to estimate the raw association between RDW and OS without adjusting for any covariates. The second statistical model (Model 2) was adjusted for clinicopathological features using findings from Model 1. The third statistical model (Model 3) consisted of a stepped entry of surgical-related covariates using findings from Model 2. To further determine whether a high RDW is an independent risk factor of OS, sensitivity analysis based on propensity score matching was conducted to confirm the independent hazards of a high RDW for OS. Low-RDW was 1:1 matched to high-RDW using Mahalanobis metric matching within a caliper width of 0.2. The relationship between RDW and OS was then re-evaluated in the PSM sample. All statistical analyses were performed using SAS statistical software (SAS Institute, Inc., Cary, NC, USA) and two-sided hypothesis testing, with a predetermined level of *P* < 0.05 considered significant.

## Results

### Patient characteristics and ROC analysis

**Fig. 1:**
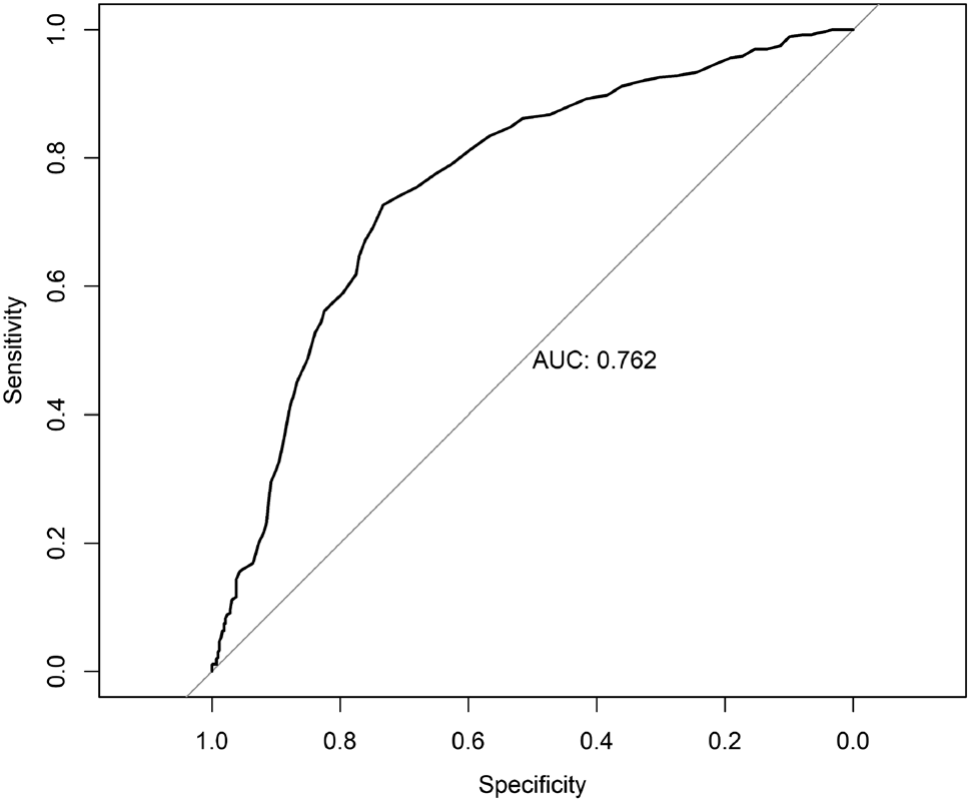
The ROC curves grouped by red-cell volume distribution width (RDW) for survival after radical surgery. *AUC* area under the curve

A total of 792 patients with pancreatic cancer treated with radical surgery were included in this study, with a median follow-up of 21.97 months (21.3, 23.63). The median RDW of all the pancreatic cancer patients was 15.0% (range 11.0–23.8%). We used ROC curve analysis to verify the power of RDW for predicting survival after radical surgery. The optimal cutoffs of the RDW for survival after PD were calculated to be 15.6% (sensitivity, 43.2%; specificity, 78.0%) (Fig. 1). The area under the curve (AUC) of RDW was 0.762 (95% CI 0.727–0.795, *P* < 0.001) for predicting survival after radical surgery. The patients were divided into two groups based on the cut-off value of the ROC curve: the low-RDW group (RDW ≤ 15.6%; *n* = 521) and the high-RDW group (RDW > 15.6%; *n* = 271).

### Correlations between the RDW and patient characteristics

Table 1 summarizes the relationship between the RDW level and clinical parameters. The average age was 57.03 ± 9.42 years in the low-RDW group and 57.63 ± 8.73 years in the high-RDW group. The percentage of male patients was 59.88% (312/521) in the low-RDW group and 58.30% (158/217) in the high-RDW group (*P* = 0.667). Fifty cases (9.60%) in the low-RDW group and 36 cases (13.28%) in the high-RDW group were classified as ASA III or upper ASA III. The patients were similar in gender, age, BMI, tumor size, blood type, location, kidney related diseases, heart related diseases, respiratory diseases, neoadjuvant therapy, postoperative chemotherapy and medical comorbidities. RDW was significantly correlated with preoperative total bilirubin (*P* < 0.001) and a history of diabetes (*P* = 0.022). The distribution of the CONUT score was as follows: 0 (*n* = 189), 1 (*n* = 104), 2 (*n* = 148), 3 (*n* = 112), 4 (*n* = 65), 5 (*n* = 87), 6 (*n* = 42), 7 (*n* = 30), and 8 (*n* = 15). The patients were divided into CONUT-normal (0 and 1; *n* = 293 [37.0%]), -light (2–4; *n* = 325 [41.0%]), -moderate (5–8; *n* = 174 [22.0%]), and -severe (9–12; *n* = 0 [0%]) groups. A significantly higher CONUT level was observed in the RDW-high group (*P* = 0.03). Pearson correlation analysis showed that RDW was associated with CONUT score (Pearson correlation *r* = 0.86, *P* < 0.001). Similarly, there was a higher proportion of high RDW in the high NLR (*P* < 0.001) and high PLR groups (*P* < 0.001). The high-RDW group had a higher proportion of patients with poorly differentiated cancer (*P* < 0.001). The RDW was found to be significantly and closely correlated with TNM stage (*P* = 0.007), especially in relation to the tumor depth (*P* < 0.001) and distant metastasis (*P* = 0.040). Among the serum tumor markers, there was a significant correlation between RDW expression and CA19-9 (Pearson correlation *r* = 0.15, *P* < 0.001), but not between any other tumor markers. The RDW was found to be positively correlated with TNM stage (Spearman correlation *r* = 0.13, *P* < 0.001).

**Table 1:** Baseline characteristics of the patients in the low-red-cell volume distribution width (RDW) group vs. those in the high-RDW group

Variable	Low-RDW (*N* = 521)	High-RDW (*N* = 271)	*P* value
Age, Mean (SD), year	57.03 (9.42)	57.63 (8.73)	0.390
BMI, Mean (SD), kg/m^2^	21.74 (2.87)	21.72 (2.96)	0.954
Tumor size, Median(IQR), cm	2.15 (1.97–2.34)	2.15 (1.99–2.34)	0.954
Sex, *N* (%)			0.667
Males	312 (59.88)	158 (58.30)	
Females	209 (40.12)	113 (41.70)	
Blood group, *N* (%)			0.862
A	175 (33.72)	83 (30.74)	
B	140 (26.97)	76 (28.15)	
AB	46 (8.86)	26 (9.63)	
O	158 (30.44)	85 (31.48)	
Diabetes mellitus, *N* (%)			0.022
No	506 (97.12)	244 (90.04)	
Yes	13 (2.50)	27 (9.96)	
Kidney related diseases, *N* (%)			0.815
No	500 (95.97)	261 (96.31)	
Yes	21 (4.03)	10 (3.69)	
Heart related diseases *N* (%)			0.751
No	469 (90.02)	242 (89.30)	
Yes	52 (9.98)	29 (10.70)	
Respiratory diseases			0.452
No	481 (92.32)	246 (90.77)	
Yes	40 (7.68)	25 (9.23)	
Family history, *N* (%)			0.458
No	505 (97.30)	266 (98.15)	
Yes	14 (2.70)	5 (1.85)	
History of surgery, *N* (%)			0.522
No	362 (69.75)	183 (67.53)	
Yes	157 (30.25)	88 (32.47)	
ASA, *N* (%)			0.114
> II	50 (9.60)	36 (13.28)	
≤ II	471 (90.40)	235 (86.72)	
CA19-9, Median(IQR), u/ml	68.42 (17.90–264.87)	148.20 (35.50–698.08)	< 0.001
CA125, Median(IQR), u/ml	15.00 (9.90–21.30)	16.20 (10.95–25.90)	0.403
CEA, Median(IQR), ng/ml	2.75 (1.78–4.44)	2.90 (1.90–4.74)	0.805
Preoperative total bilirubin, Median(IQR), umol	59.10 (38.30–85.50)	80.65 (55.30–120.80)	< 0.001
Location, *N* (%)			0.158
Head of pancreas	404 (77.54)	213 (78.60)	
Neck of pancreas	11 (2.11)	4 (1.48)	
Body of pancreas	39 (7.49)	15 (5.54)	
Tail of pancreas	65 (12.48)	38 (14.02)	
Total of pancreas	2 (0.38)	1 (0.37)	
Grade, *N* (%)			< 0.001
Poor	180 (34.55)	142 (52.40)	
Moderate	246 (47.22)	103 (38.01)	
Well	95 (18.23)	26 (9.59)	
Depth of tumor, *N* (%)			< 0.001
T1	231 (47.43)	81 (31.64)	
T2	218 (44.76)	146 (57.03)	
T3	37 (7.60)	29 (11.33)	
T4	1 (0.21)	0 (0.00)	
Lymph node metastasis, *N* (%)			0.145
N0	343 (70.43)	163 (63.67)	
N1	120 (24.64)	80 (31.25)	
N2	24 (4.93)	13 (5.08)	
Distance metastasis, *N* (%)			0.040
M0	481 (99.59)	251 (98.05)	
M1	2 (0.41)	5 (1.95)	
pStage, *N* (%)			0.007
IA	171 (35.11)	60 (23.44)	
IB	145 (29.77)	81 (31.64)	
IIA	26 (5.34)	19 (7.42)	
IIB	118 (24.23)	79 (30.86)	
III	25 (5.13)	12 (4.69)	
IV	2 (0.41)	5 (1.95)	
CONUT, *N* (%)			< 0.001
Normal	291 (55.85)	2 (0.74)	
Mild	220 (42.23)	105 (38.75)	
Moderate	10 (1.92)	164 (60.52)	
Severe	0 (0.00)	0 (0.00)	
NLR			< 0.001
< 2.8	260 (49.90)	99 (36.53)	
≥ 2.8	261 (50.10)	172 (63.47)	
PLR			< 0.001
< 186	279 (53.55)	84 (31.00)	
≥ 186	242 (46.45)	187 (69.00)	
Neoadjuvant therapy			0.420
No	478 (91.75)	253 (93.36)	
Yes	43 (8.25)	18 (6.64)	
Postoperative chemotherapy			0.093
No	2 (0.38)	4 (1.48)	
Yes	519 (99.62)	267 (98.52)	

*BMI* body mass index, *ASA* American society of anesthesiologists, *CA19-9* carcinoembryonic antigen 19-9, *CA125* carcinoembryonic antigen 125, *CEA* carcinoembryonic antigen, *CONUT* controlling nutritional status, *SD* standard deviation, *IQR* interquartile range

### Correlations between RDW and intraoperative characteristics

A high RDW was associated with a longer surgical time (mean, 374 vs 342 min, *P* < 0.001), decreased R0 removal rate (75.28% vs 85.03%, *P* < 0.001), more blood loss (mean, 452 vs 346 mL, *P* = 0.011), and increased intraoperative blood transfusion volume (mean, 1.87 vs 1.43 U, *P* < 0.001; Table 2). There was no significant difference in the number of lymph nodes cleared between the high and low RDW groups (*P* = 0.411).

**Table 2:** Intraoperative factors in the low-red-cell volume distribution width (RDW) group vs. those in the high-RDW group

Variable	Low-RDW (*N* = 521)	High-RDW (*N* = 271)	*P* value
Duration of surgery, Mean (SD), min	342.15 (99.72)	374.37 (113.12)	< 0.001
Intraoperative bleeding, Mean (SD), ml	346.15 (49.25)	451.73 (40.65)	0.011
Red blood cell transfusion, Mean (SD), U	1.43 (3.09)	1.87 (2.40)	< 0.001
R state, *N* (%)			< 0.001
R0	443 (85.03)	204 (75.28)	
R1	78 (14.97)	67 (24.72)	
Lymph node dissection, median(IQR)	15 (13–25)	16 (13–28)	0.411

*SD* standard deviation, *IQR* interquartile range

### Correlations between RDW and postoperative characteristics

Table 3 shows the postoperative factors. The incidence of complications was similar in the two groups (25.53% *vs* 28.41%, *P* = 0.383). Specifically, there were no significant differences in gastrointestinal fistula, bile leakage, liver failure, renal failure, or delayed gastric emptying. However, the pulmonary infection rate was significantly higher in the high-RDW group than in the low-RDW group (2.95% vs 0.77%, *P* = 0.017). The rate of postoperative bleeding was also significantly higher in the high-RDW group (11.07% vs 6.74%, *P* = 0.036). The high-RDW group had a higher rate of postoperative pancreatic fistula (16.24% vs 11.71%, *P* = 0.043). The positive lymph node rate was significantly higher in the high-RDW group than in the low-RDW group (34.32% vs 18.81%, *P* = 0.008). There was no difference in the median length of hospital stay after surgery (21 vs 21 days, *P* = 0.113). The reoperation rate in the high-RDW group was significantly higher (3.32% vs 0.77%, *P* = 0.007). Both 30-day mortality (5.90% vs 0.19%, *P* < 0.001) and 90-day mortality (12.92% vs 0.77%, *P* < 0.001) were significantly higher in the high-RDW group.

**Table 3:** Short-term postoperative results in the low-red-cell volume distribution width (RDW) group vs. those in the high-RDW group

Variable	Low-RDW (*N* = 521)	High-RDW (*N* = 271)	*P* value
Postoperative hospital stay, Median (IQR), day	21.00 (16.00–28.00)	21.00 (16.00–26.00)	0.113
Positive lymph node, *N* (%)	98 (18.81)	93 (34.32)	0.008
Aggregate complications, *N* (%)	133 (25.53)	77 (28.41)	0.383
Renal failure, *N* (%)	4 (0.77)	1 (0.37)	0.076
Pulmonary complications, *N* (%)	4 (0.77)	8 (2.95)	0.017
Hepatic failure, *N* (%)	2 (0.39)	0 (0.00)	0.306
Gastrointestinal fistula, *N* (%)	2 (0.39)	0 (0.00)	0.306
Biliary leakage, *N* (%)	2 (0.39)	0 (0.00)	0.306
Postpancreatectomy Hemorrhage, *N* (%)	35 (6.74)	30 (11.07)	0.036
Pancreatic fistula, *N* (%)	61 (11.71)	44 (16.24)	0.043
Delayed gastric emptying of grade B/C, *N* (%)	130 (24.95)	69 (25.46)	0.102
Reoperation, *N* (%)	4 (0.77)	9 (3.32)	0.007
30-Day mortality, *N* (%)	1 (0.19)	16 (5.90)	< 0.001
90-Day mortality, *N* (%)	4 (0.77)	35 (12.92)	< 0.001

*SD* standard deviation, *IQR* interquartile range

### RDW is an independent prognostic marker for pancreatic cancer patients

**Fig. 2:**
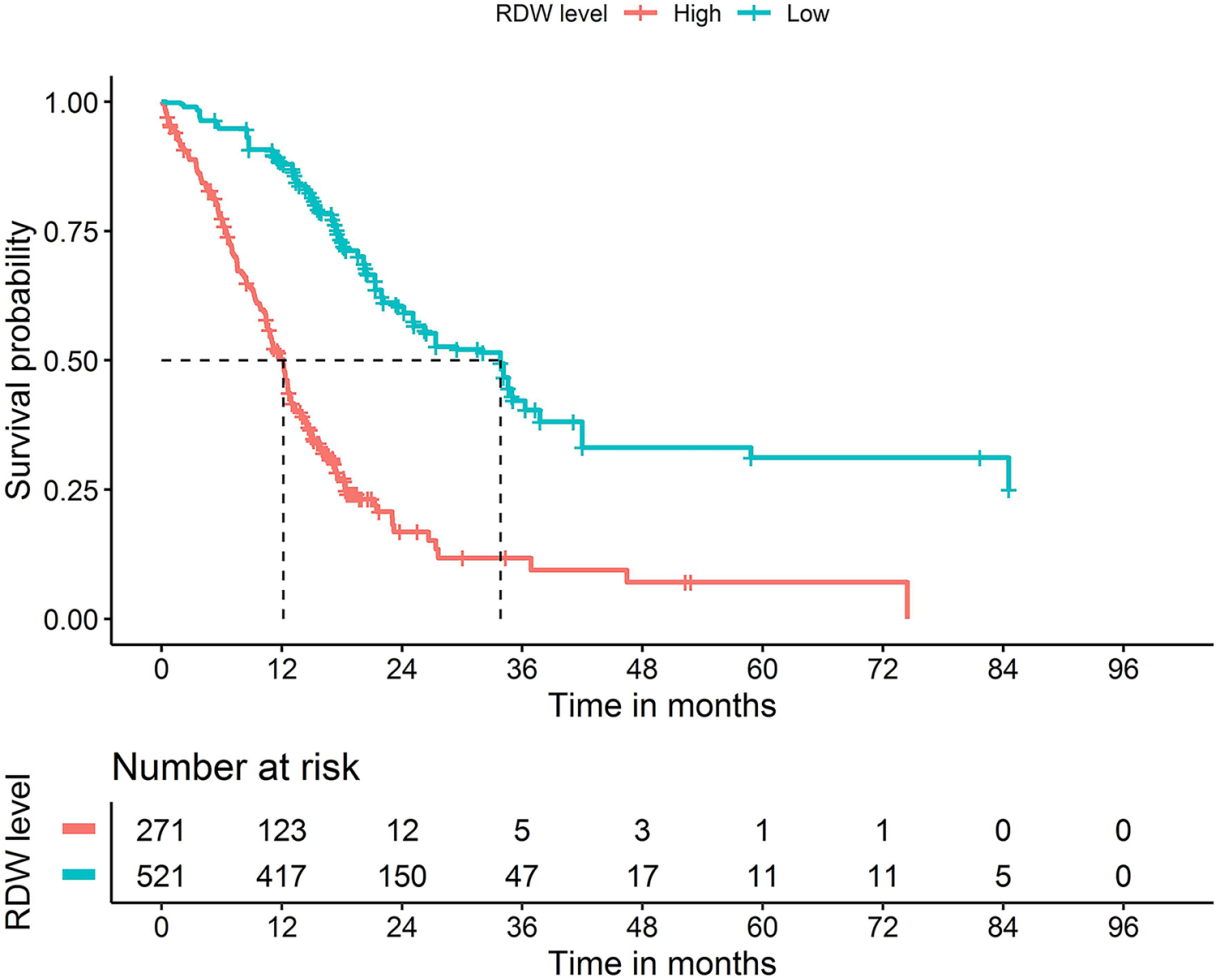
Kaplan–Meier curves for the overall survival (OS) of patients who underwent radical surgery, based on red-cell volume distribution width (RDW) (*P* < 0.001)

Table 4 shows that in Model 1, a high RDW was associated with OS with a hazard ratio of 3.664 (95% CI 2.931–4.580, *P* < 0.001) without adjusting for any covariate, whereas in Model 2, after adjusting for age, gender, diabetes mellitus, ASA, BMI, preoperative CA19-9, preoperative bilirubin, pH, preoperative albumin, preoperative r-GT, preoperative cholesterol, and preoperative white blood cell, the high RDW was still indicated as an independent risk factor of OS (HR, 2.659; 95% CI 1.445–4.891, *P* = 0.002). When the intraoperative covariates were adjusted further, the significant risk effect of high-RDW to OS was robust (Model 3, HR, 2.544; 95% CI 1.313–4.930, *P* = 0.006). Moreover, in the PSM model, 217 patients in the high-RDW group were matched with 217 patients in the low-RDW group (the PSM is shown in the Online resource 2), and a high RDW remained an independent risk factor of OS (PSM Model, HR, 3.230; 95% CI 2.427–4.297, *P* < 0.001). All four models showed that RDW was an independent prognostic marker predictive of poor OS for pancreatic cancer patients after surgery. Univariate and multivariate analysis showed that cholesterol (HR 0.849, 95% CI 0.766–0.941, *P* = 0.002), RDW (HR 2.661, 95% CI 2.014–3.515, *P* < 0.001), CONUT (Mild: Normal, HR 1.123, 95% CI 1.032–1.889, *P* = 0.231; Moderate: Mild, HR 3.010, 95% CI 2.223–3.987, *P* < 0.001; Severe: Moderate, HR 4.598, 95% CI 3.058–6.114, *P* = 0.011.), and AJCC stage (II:I, HR 1.803, 95% CI 1.364–2.385, *P* < 0.001; III:II, HR 2.923, 95% CI 1.967–4.343, *P* < 0.001; IV:III, HR 34.009, 95% CI 4.608–251.019, *P* = 0.001.) were independent prognostic indices for OS (Online Resource 3). The Kaplan–Meier survival curve also suggested that a high RDW was associated with low OS, and that the difference in survival rates between the high-RDW group and the low-RDW group was significant (*P* < 0.001, Fig. 2).

**Table 4:** Risk factors analysis of the red-cell volume distribution width (RDW) as a prognostic factor for pancreatic cancer

	HR (95% CI)	Chi-square	*P*^a^ value
Model 1^b^	3.664 (2.931–4.580)	130.19	< 0.001
Model 2^c^	2.659 (1.445–4.891)	9.89	0.002
Model 3^d^	2.544 (1.313–4.930)	7.65	0.006
PSM Model	3.230 (2.427–4.297)	64.72	< 0.001

The PSM Model consisted of age, location, gender, diabetes mellitus, ASA, BMI, preoperative CA19-9, preoperative bilirubin, pH, preoperative albumin, preoperative r-GT, preoperative cholesterol, preoperative white blood cell, intraoperative bleeding, red blood cell transfusion, lymph node dissection, complication, R state, pancreas texture, grade, TNM stage, complications, surgery history, tumor size, preoperative ALP, preoperative biliary drainage, duration of surgery, 30 days unplanned readmission, with CONUT and other parameters being covariates

^a^The hazards ratio of being high RDW group

^b^The Raw model without adjusting for any covariate

^c^Multivariate Cox hazard risk model with clinical preoperative characteristics including age, gender, diabetes mellitus, ASA, BMI, preoperative CA19-9, preoperative bilirubin, pH, preoperative albumin, preoperative r-GT, preoperative cholesterol, and preoperative white blood cell being adjusted on the basis of model 1

^d^Multivariate Cox hazard risk model with intraoperative variables, including location, intraoperative bleeding, red blood cell transfusion, lymph node dissection, complication, R state, pancreas texture being further adjusted on the basis of model 2

## Discussion

The incidence of pancreatic cancer has been increasing in recent years [1]. Despite the improvements in surgical facilities and techniques, the survival rate of pancreatic cancer patients after radical surgery is still relatively poor. Therefore, it is important to identify preoperatively the factors that may predict postoperative survival. Currently, there is still no suitable blood test or assay that can be done preoperatively to predict morbidity and mortality.

The RDW, which is one of the standard parameters in the routine reports of all automated blood analyzers, has attracted the attention of tumor researchers. RDW is the width of the frequency distribution curve of red blood cell volume (one SD) divided by the average red blood cell volume [24]. A meta-analysis suggested that RDW may be a suitable prognostic marker in cancer patients [25]. Although a recent study on pancreatic cancer found that elevated RDW levels in patients with pancreatic masses may indicate malignancy [26], no study has shown that RDW can be used as a prognostic marker for pancreatic cancer. In this study, the ROC curve analysis was used to verify the predictive power of the RDW for survival after surgery. The patients were divided into two groups by ROC value: a low-RDW group and a high-RDW group. Although there was no significant difference in postoperative complications between the groups, the postoperative bleeding rate, postoperative pancreatic fistula rate, and reoperation rate were higher in the high-RDW group than in the low-RDW group. The high-RDW group also had higher 30- and 90-day mortality rates after surgery. Thus, among pancreatic cancer patients undergoing surgery, the high-RDW group had a worse short-term prognosis than the low-RDW group. In fact, the patients with a high RDW had more difficulties during the operation, requiring longer operation times, with more blood loss and a lower R0 resection rate. According to the Kaplan–Meier curves of OS, the prognosis of the high-RDW group was significantly worse than that of low-RDW group. This indicated that a high RDW predicted not only poor short-term prognosis but also poor long-term prognosis. On this basis, we conducted a risk analysis on the factors influencing patient survival and confirmed that the RDW is an independent factor influencing the postoperative survival of patients with pancreatic cancer. To our knowledge, ours is the first institution to report that the RDW can be used as an independent prognostic factor for patients with pancreatic cancer undergoing radical surgery. Thus, the preoperative evaluation of RDW may be critical for determining the timing of surgery. In the clinical setting, it is possible to reduce the preoperative RDW through perioperative treatment, which improves the postoperative survival rate of pancreatic cancer patients. Patients with a high RDW may be in a later stage of the disease. Even in patients with borderline status, such as those with a borderline resectable tumor, the duration of neoadjuvant therapy and the timing of surgery can be determined by measuring the RDW. However, more meaningful studies in larger populations are needed before its potential can be fully realized in clinical settings.

The mechanisms underlying the effect of RDW on pancreatic cancer are still unknown, but more studies have shown that the RDW promotes the development of cancer through inflammation of the microenvironment. The CONUT score, which is considered representative of immune-nutritional status, was calculated from the serum albumin concentration, total peripheral lymphocyte count, and total cholesterol level [12]. Based on the significant correlation between the RDW and CONUT score in this study, the RDW may be related to immune-nutritional status, which is consistent with other reports [27]. Furthermore, a high RDW was associated with higher rates of diabetes mellitus and higher preoperative total bilirubin. Patients with diabetes and high bilirubin tend to have poor nutritional status. This also reflects the poor nutritional status of patients in the high-RDW group. The RDW has been shown to be affected by increased systemic inflammation, which not only impedes the survival of red blood cells but also distorts cell membranes [28]. Lippi et al. identified a relationship between RDW and blood markers of inflammation such as highly sensitive c-reactive protein and erythrocyte sedimentation rate [29]. The tumor microenvironment is in a chronic inflammatory state, and tumor cells secrete a variety of inflammatory mediators, making RDW parameters suitable tumor-related markers [30, 31]. In fact, higher RDW levels are associated with increased activity in several inflammatory diseases [32–34]. RDW has been shown to be strongly associated with cancer progression through systemic inflammatory response, particularly in lung, breast, and colon cancers [14, 35, 36]. There are also a number of hypotheses about the causes of increased RDW, including the possible origin of bone marrow suppression [30], and that chronic inflammation leads to an iron metabolism disorder that reduces the production of erythropoietin, which may impair hematopoietic function [37, 38]. Therefore, although the mechanism underlying the influence of RDW on the postoperative survival of pancreatic cancer patients needs to be studied further, the association between RDW and nutritional immunity should be a focus of attention.

Considering the association between preoperative RDW and nutrition, the RDW can be used as a prognostic factor for radical surgery. Findings suggest that preoperative nutritional intervention may improve the postoperative survival of patients with pancreatic cancer. A series of studies have shown that nutritional interventions before treatment can improve the survival of patients with esophageal cancer [39] and those with head and neck cancer [40, 41]. Although it is not known whether nutritional intervention can prolong the survival of pancreatic cancer patients, it has been reported that nutritional intervention can mitigate the weight loss and physical strength loss of patients with advanced non-small cell lung cancer [42]. The possibility of improving preoperative nutritional status and thereby improving postoperative prognosis needs to be verified. A means of intervening in the preoperative nutrition of patients with pancreatic cancer must also be investigated.

A high CA19-9 level can also indicate a patient’s cancer level and help surgeons decide how and when to perform surgery. A meta-analysis showed that CA19-9 levels play an important role in the identification and diagnosis of early pancreatic cancer [43]. In this study, patients with a high CA19-9 concentration had a high RDW, and Pearson’s correlation analysis showed a positive correlation between RDW and CA19-9. This also indicates that RDW reflects the prognosis of pancreatic cancer patients to some extent. Moreover, we found that patients with higher TNM stage also had a higher RDW, and Pearson correlation coefficient analysis showed that RDW was positively correlated with TNM stage. The high-RDW group even included a higher proportion of patients with poorly differentiated pancreatic cancer. Given this, it is not surprising that the higher the value of RDW, the worse the short-term and long-term survival status of patients. The same results have been reported for patients with colorectal, esophageal, lung, and endometrial cancers [14, 44–46]. Therefore, the RDW may be used as a marker to predict the degree of malignancy of tumors. Because it is an indicator in blood routine testing, it can be obtained simply and noninvasively, which has a positive effect on predicting the prognosis of patients undergoing surgery for pancreatic cancer.

This study had some limitations. First, as with most retrospective design studies, the data collection may have been biased by recall bias. In our study, the data were extracted through an electronic database constructed since 2012 to collect data prospectively in our center. Thus, the data quality can be guaranteed, with a reliable result based on the high quality of our research data. Second, the lack of research on the mechanism prevented us from fully understanding the value of the RDW for pancreatic cancer. However, we identified that a high RDW level had an obviously significant effect on OS, which enriched the evidence that the RDW is associated with the long-term prognosis of pancreatic cancer patients. These results provide rich information for laboratory research to further investigate the relationship between the RDW and the prognosis of pancreatic cancer patients. Third, the follow-up period may not have been long enough and is still ongoing. We will have more robust information for our future studies. Overall, we expect our findings to be validated in further multicenter studies with larger sample sizes and less heterogeneity.

## Conclusion

In summary, the results of this study showed that the preoperative RDW value, which was associated with nutritional immunity, was a good predictor of the prognosis of pancreatic cancer patients undergoing radical surgery. The RDW was also positively correlated with CA19-9 and tumor stage. As our findings were based on a single-center, small-sample study, further well-designed prospective studies should be conducted to confirm the conclusions.

## Supplementary Information

Below is the link to the electronic supplementary material.

Supplementary file1 (DOCX 46 KB)
