# Supplementary material for: Clinical importance of preoperative red-cell volume distribution width as a prognostic marker in patients undergoing radical surgery for pancreatic cancer
Source: Surg Today. 2021 Sep 15;52(3):465–74. doi: 10.1007/s00595-021-02374-7 (PMC8873122; doi:10.1007/s00595-021-02374-7)
Supplement: Supplementary file 1 — Supplementary file1 (DOCX 46 KB) [file 595_2021_2374_MOESM1_ESM.docx]

**Online Resources**

**Online Resource 1** The CONUT scoring system

| **Parameters** | **Normal** | **Mild** | **Moderate** | **Severe** |
| --- | --- | --- | --- | --- |
| Serum albumin [g/ml] | ≥3.5 | 3.0-3.4 | 2.5-2.9 | < 2.50 |
| (Score) | 0 | 2 | 4 | 6 |
| Total lymphocyte count | ≥1600 | 1200-1599 | 800-1199 | < 800 |
| (Score) | 0 | 1 | 2 | 3 |
| Total cholesterol [mg/dl] | ≥180 | 140-179 | 100-139 | < 100 |
| (Score) | 0 | 1 | 2 | 3 |
| Total score | 0-1 | 2-4 | 5-8 | 9-12 |
| Dysnutritional states | Normal | Mild | Moderate | Severe |

**Abbreviation:** CONUT, controlling nutritional status.

**Online Resource 2** The baseline characteristics of before and after PSM between Low-RDW and High-RDW group

| **Parameters** | **Before PSM** | | |  | **After PSM** | | | |
| --- | --- | --- | --- | --- | --- | --- | --- | --- |
|  | **Low-RDW**  **(N=516)** | **High-RDW**  **(N=269)** | **P Value** |  | **Low-RDW**  **(N=217)** | **High-RDW**  **(N=217)** | **P Value** | **SMD** |
| Sex N(%) (Females) |  |  | 0.667 |  |  |  | 0.635 | 0.044 |
| Male | 309(59.88) | 156(57.99) |  |  | 122(56.22) | 127(58.53) |  |  |
| Females | 207(40.12) | 113(42.00) |  |  | 95(43.78) | 90(41.47) |  |  |
| Age Mean(SD), year | 57.03(9.42) | 57.63(8.73) | 0.390 |  | 57.77(9.52) | 57.43(8.64) | 0.690 | 0.037 |
| Blood group, N(%) |  |  | 0.862 |  |  |  | 0.807 | 0.017 |
| A | 174 (33.7) | 82(30.5) |  |  | 72(33.18) | 64(29.49) |  |  |
| AB | 46 (8.9) | 26(9.7) |  |  | 16(7.37) | 17(7.83) |  |  |
| B | 137 (26.6) | 75(27.9) |  |  | 57(26.27) | 65(29.95) |  |  |
| O | 157 (30.4) | 85(31.6) |  |  | 72(33.18) | 71(32.72) |  |  |
| Diabetes mellitus N(%) | 13 (2.5) | 27(10.0) | 0.192 |  | 9(4.15) | 5(2.30) | 0.033 | 0.198 |
| Family history, N(%) | 14(2.71) | 5(1.86) | 0.458 |  | 10(4.61) | 5(2.30) | 0.184 | 0.103 |
| History of Surgery, N(%) | 156(30.23) | 87(32.34) | 0.522 |  | 70(32.26) | 76(35.02) | 0.591 | 0.050 |
| Tumor size, Mean(SD), cm | 2.37(1.26) | 2.64(1.36) | 0.006 |  | 2.44(1.29) | 2.66(1.41) | 0.079 | 0.106 |
| ALP, Median [IQR] U/L | 272.50(130.00~447.00) | 326.00(165.00~555.00) | 0.010 |  | 257.00(130.00~422.00) | 312.50(153.00~549.50) | 0.069 | 0.112 |
| r-GT, Median [IQR], U/L | 435.50(139.00~782.00) | 379.00(154.00~715.00) | 0.435 |  | 405.50(114.00~765.00) | 392.00(149.50~732.50) | 0.844 | 0.019 |
| [Cholesterol](javascript:;), Median [IQR], mmol/L | 4.82(4.07~5.94) | 3.43(2.87~3.99) | <0.001 |  | 4.45(3.84~5.27) | 3.54(2.90~4.18) | <0.001 | 0.921 |
| [Triglyceride](javascript:;), Median [IQR], mmol/L | 1.53(1.02~2.16) | 1.61(1.01~2.35) | 0.880 |  | 1.56(1.03~2.19) | 1.64(1.05~2.30) | 0.848 | 0.087 |
| [Albumin](javascript:;), Median [IQR], g/L | 38.30(36.60~42.00) | 32.45(29.90~34.65) | <0.001 |  | 37.40(35.60~40.00) | 32.70(29.90~34.90) | <0.001 | 0.239 |
| [White](javascript:;) [Blood](javascript:;) [Cell](javascript:;), Median [IQR],10^9^/L | 5.43(4.48~7.02) | 5.72(4.40~7.26) | 0.098 |  | 5.39(4.47~6.72) | 5.61(4.42~6.98) | 0.155 | 0.105 |
| Preoperative biliary drainage, N(%) | 203(39.34) | 108(40.14) | 0.808 |  | 80(36.87) | 87(40.09) | 0.497 | 0.063 |
| ASA, N(%)(≤II) | 471 (90.40) | 235(86.72) | 0.114 |  | 167(76.96) | 170(78.34) | 0.574 | 0.052 |
| CONUT N(%) |  |  | <0.001 |  |  |  | <0.001 | 0.124 |
| Normal | 291(55.85) | 2(0.74) |  |  | 88(40.55) | 2(0.92) |  |  |
| Mild | 220(42.23) | 105(38.75) |  |  | 119(54.84) | 102(47.00) |  |  |
| Moderate | 10(1.92) | 164(60.52) |  |  | 10(4.61) | 113(52.07) |  |  |
| Severe | 0(0.00) | 0(0.00) |  |  | 0(0.00) | 0(0.00) |  |  |
| Location, N(%) |  |  | 0.235 |  |  |  | 0.912 | 0.023 |
| Head of pancreas | 404(77.54) | 213(78.60) |  |  | 170(78.34) | 174(80.18) |  |  |
| Neck of pancreas | 11(2.11) | 4(1.48) |  |  | 3(1.38) | 3(1.38) |  |  |
| Body of pancreas | 39(7.49) | 15(5.54) |  |  | 13(5.99) | 12(5.53) |  |  |
| Tail of pancreas | 65(12.48) | 38(14.02) |  |  | 30(13.82) | 27(12.44) |  |  |
| Total of pancreas | 2(0.38) | 1(0.37) |  |  | 1(0.46) | 1(0.46) |  |  |
| Grade, N(%) |  |  | 0.012 |  |  |  | 0.436 | 0.028 |
| Poor | 180(34.55) | 142(52.40) |  |  | 93(42.86) | 94(43.31) |  |  |
| Moderate | 246(47.22) | 103(38.01) |  |  | 95(43.78) | 97(44.70) |  |  |
| Well | 95(18.23) | 26(9.59) |  |  | 29(13.36) | 26(11.98) |  |  |
| pStage, N(%) |  |  | 0.007 |  |  |  | 0.152 | 0.084 |
| IA | 171(35.11) | 60(23.44) |  |  | 71(32.72) | 50(23.04) |  |  |
| IB | 145(29.77) | 81(31.64) |  |  | 76(35.02) | 67(30.88) |  |  |
| IIA | 26(5.34) | 19(7.42) |  |  | 7(3.23) | 15(6.91) |  |  |
| IIB | 118(24.23) | 79(30.86) |  |  | 53(24.42) | 69(31.80) |  |  |
| III | 25(5.13) | 12(4.69) |  |  | 8(3.69) | 12(5.53) |  |  |
| IV | 2(0.41) | 5(1.95) |  |  | 1(0.46) | 4(1.84) |  |  |
| 30 days unplanned readmission N(%) (Yes) | 28(5.37) | 10(3.69) | 0.293 |  | 16(7.37) | 8(3.69) | 0.093 | 0.097 |
| Duration of surgery, Median [IQR], min | 330.00(260.00~400.00) | 380.00(300.00~450.00) | <0.001 |  | 340.00(270.00~420.00) | 378.00(300.00~444.00) | 0.057 | 0.117 |
| Intraoperative bleeding, Median [IQR], ml | 300.00(100.00~500.00) | 300.00(100.00~600.00) | 0.111 |  | 300.00(100.00~500.00) | 350.00(100.00~600.00) | 0.345 | 0.088 |
| Red blood cell transfusion, Median [IQR], U | 0.00(0.00~2.00) | 0.00(0.00~4.00) | 0.043 |  | 0.00(0.00~3.00) | 0.00(0.00~4.00) | 0.524 | 0.060 |
| Lymph node dissection, Median(IQR) | 13(1.22) | 13(0.52) | 0.044 |  | 13(0.36) | 13(0.75) | 0.432 | 0.020 |
| R state, N(%)(R1) | 78(14.97) | 67(24.72) | 0.001 |  | 45(20.74) | 56(25.81) | 0.215 | 0.106 |
| Pancreas texture, N (%) |  |  | <0.001 |  |  |  | 0.798 | 0.108 |
| Soft | 260(50.4) | 93(34.6) |  |  | 103(47.47) | 81(37.33) |  |  |
| Hard | 156(30.2) | 124(46.1) |  |  | 76(35.02) | 96(44.24) |  |  |
| Moderate | 98(19.0) | 51(19.0) |  |  | 38(17.51) | 40(18.43) |  |  |
| Aggregate complications, N(%)(Yes) | 133(25.53) | 77(28.41) | 0.383 |  | 63(29.03) | 65(29.95) | 0.528 | 0.059 |
| Renal failure | 4(0.77) | 1(0.37) | 0.076 |  | 3(1.38) | 1(0.46) | 0.319 | 0.093 |
| Pulmonary complications | 4(0.77) | 8(2.95) | 0.017 |  | 3(1.38) | 7(3.23) | 0.206 | 0.109 |
| Hepatic failure | 2(0.39) | 0(0.00) | 0.306 |  | 1(0.46) | 1(0.46) | 0.995 | 0.001 |
| Infection | 21(4.05) | 18(6.64) | 0.110 |  | 10(4.61) | 18(8.29) | 0.124 | 0.044 |
| Gastrointestinal fistula | 2(0.39) | 0(0.00) | 0.306 |  | 1(0.46) | 0(0.00) | 0.315 | 0.094 |
| Biliary leakage | 2(0.39) | 0(0.00) | 0.306 |  | 0(0.00) | 0(0.00) | NaN | 0.001 |
| Postpancreatectomy hemorrhage | 35(6.74) | 30(11.07) | 0.036 |  | 19(8.76) | 26(11.98) | 0.285 | 0.100 |
| Pancreatic fistula | 61(11.71) | 44(16.24) | 0.043 |  | 32(14.75) | 26(11.98) | 0.379 | 0.082 |
| Delayed gastric emptying of grade B/C | 130(24.95) | 69(25.46) | 0.102 |  | 62(28.57) | 64(29.49) | 0.832 | 0.020 |
| Reoperation N(%) | 4(0.77) | 9(3.32) | 0.007 |  | 3(1.38) | 8(3.69) | 0.127 | 0.093 |
| 30-Day mortality, N(%) | 1(0.2) | 16(5.90) | <0.001 |  | 1(0.46) | 12(5.53) | 0.001 | 0.292 |
| 90-Day mortality, N(%) | 4(0.77) | 35(12.92) | <0.001 |  | 3(1.38) | 30(13.82) | <0.001 | 0.443 |

**Abbreviation:** PSM, Propensity Score Matching; SMD, STD Mean Difference; ALP, alkaline phosphatase; r-GT, gamma-glutamyl transpeptidase; ASA, American society of anesthesiologists; SD, standard derivation; IQR interquartile range.

**Online Resource 3** The univariate and multivariate Cox model to evaluate the prognostic factors for pancreatic cancer.

| **Parameter** | **Univariate analysis** | |  | **Multivariate analysis** | |
| --- | --- | --- | --- | --- | --- |
|  | **HR(95%CI)** | **P value** |  | **HR(95%CI)** | **P value** |
| Age | 1.006(0.994~1.018) | 0.325 |  |  |  |
| BMI | 0.949(0.908~0.991) | 0.018 |  |  |  |
| Female | 0.883(0.706~1.104) | 0.274 |  |  |  |
| Tumor Size | 0.949(0.908~0.991) | 0.018 |  |  |  |
| ALP | 1.000(0.999~1.000) | 0.019 |  |  |  |
| r-GT | 1.000(1.000~1.000) | 0.122 |  |  |  |
| [Cholesterol](javascript:;) | 0.677(0.614~0.747) | <0.001 |  | 0.849(0.766~0.941) | 0.002 |
| [Triglyceride](javascript:;) | 1.047(0.954~1.148) | 0.333 |  |  |  |
| [Albumin](javascript:;) | 0.892(0.871~0.912) | <0.001 |  |  |  |
| White Blood Cell | 0.981(0.944~1.020) | 0.346 |  |  |  |
| Preoperative biliary drainage | 0.992(0.788~1.249) | 0.946 |  |  |  |
| CA19-9 | 1.000(1.000~1.002) | <0.001 |  |  |  |
| CA125 | 1.005(1.001~1.009) | 0.009 |  |  |  |
| CEA | 1.231(1.005~1.325) | <0.001 |  |  |  |
| Operation Time | 1.002(1.001~1.003) | <0.001 |  |  |  |
| Intraoperative bleeding | 1.000(1.000~1.000) | 0.011 |  |  |  |
| High RDW | 3.664(2.931~4.580) | <0.001 |  | 2.661(2.014~3.515) | <0.001 |
| CONUT |  |  |  |  |  |
| Normal | Reference |  |  | Reference |  |
| Mild | 1.258(1.068~1.982) | 0.022 |  | 1.123(1.032~1.889) | 0.231 |
| Moderate | 3.151(2.288~4.339) | <0.001 |  | 3.010(2.223~3.987) | <0.001 |
| Severe | 5.073(3.623~7.104) | <0.001 |  | 4.598(3.058~6.114) | 0.011 |
| NLR |  |  |  |  |  |
| <2.8 | Reference |  |  |  |  |
| ≥2.8 | 1.724(1.340~2.218) | 0.026 |  |  |  |
| PLR |  |  |  |  |  |
| <186 | Reference |  |  |  |  |
| ≥186 | 1.732(1.350~2.222) | 0.045 |  |  |  |
| Blood type |  |  |  |  |  |
| A | Reference |  |  |  |  |
| AB | 1.120(0.765~1.640) | 0.561 |  |  |  |
| B | 0.843(0.631~1.127) | 0.250 |  |  |  |
| O | 0.995(0.757~1.307) | 0.971 |  |  |  |
| Diabetes mellitus (Yes) | 0.553(0.285~1.073) | 0.080 |  |  |  |
| Family history (Yes) | 1.006(0.551~1.838) | 0.984 |  |  |  |
| History of Surgery (Yes) | 1.006(0.793~1.276) | 0.962 |  |  |  |
| ASA |  |  |  |  |  |
| ≤II | Reference |  |  |  |  |
| >II | 0.627(0.450~0.874) | 0.006 |  |  |  |
| AJCC |  |  |  |  |  |
| I | Reference |  |  | Reference |  |
| II | 1.842(1.443~2.352) | <0.001 |  | 1.803(1.364~2.385) | <0.001 |
| III | 2.341(1.578~3.475) | <0.001 |  | 2.923(1.967~4.343) | <0.001 |
| IV | 24.692(3.377~180.561) | 0.002 |  | 34.009(4.608~251.019) | 0.001 |
| Pancreas texture |  |  |  |  |  |
| Soft | Reference |  |  |  |  |
| Hard | 1.742(1.361~2.232) | <0.001 |  |  |  |
| Moderate | 1.265(0.925~1.730) | 0.141 |  |  |  |
| Aggregate complications (Yes) | 1.244(0.976~1.585) | 0.079 |  |  |  |
| Readmission | 0.867(0.532~1.414) | 0.568 |  |  |  |
| Reoperation | 2.049(1.016~4.134) | 0.045 |  |  |  |

**Abbreviation:** BMI, body mass index; ALP, alkaline phosphatase; r-GT, gamma-glutamyl transpeptidase; CA19-9, carcinoembryonic antigen 19-9; CA125, carcinoembryonic antigen 125; CEA, carcinoembryonic antigen; ASA, American society of anesthesiologists.
